# The perception of diabetes risk in minor children among parents with type 2 diabetes mellitus: a qualitative study

**DOI:** 10.3389/fpubh.2026.1846450

**Published:** 2026-06-04

**Authors:** Jingjing Wang, Songmei Cao, Qiaoyan Liu, Xiangjie Shen, Houjuan Zu, San Fan

**Affiliations:** 1 Department of Endocrinology and Metabolism, Affiliated Hospital of Jiangsu University, Zhenjiang, Jiangsu, China; 2 Department of Nursing, Affiliated Hospital of Jiangsu University, Zhenjiang, Jiangsu, China

**Keywords:** offspring, parents, perceived risk of disease, qualitative research, type 2 diabetes

## Abstract

**Background:**

Parental perceptions of diabetes risk in minor children are crucial for preventing or delaying the onset of type 2 diabetes mellitus in children of parents living with the condition. However, research on this topic remains limited. In this context, the present study aimed to examine parental risk perceptions concerning pediatric type 2 diabetes to provide evidence-based guidance for developing early intervention strategies for diabetes prevention.

**Method:**

Purposive sampling was used to recruit 16 parents with type 2 diabetes mellitus who had minor children. Participants were both outpatient and inpatient attendees of the Department of Endocrinology and Metabolism at a tertiary Grade A hospital in Zhenjiang. The recruitment period spanned from June to August 2025. A phenomenological research design was employed, and semi-structured interviews were conducted. Data analysis followed Colaizzi's seven-step analytical framework.

**Results:**

Parents' perceptions of type 2 diabetes risk in their minor children were categorized into three core themes: cognitive biases in the perception of genetic risk (including genetic determinism, the concept of acquired illness leads to cognitive impairment, and gender biases in risk perception), the double-edged sword of behavioral changes following risk perception (encompassing internalization of reasonable health behaviors and governance of excessive health behaviors), and dilemma in health behavior intervention following risk perception (such as intergenerational conflict, conflict between academic load and health maintenance, scarcity of health management information, and game of health sovereignty).

**Conclusion:**

Healthcare professionals should assist parents with type 2 diabetes mellitus in developing accurate perceptions of genetic risk, enhancing their comprehensive understanding of pediatric diabetes risks, strengthening family-based support systems, and facilitating the implementation of targeted preventive behavioral interventions.

## 1 Introduction

The global prevalence of type 2 diabetes mellitus continues to rise, with a notable shift toward younger populations. Specifically, the increasing incidence of type 2 diabetes among children and adolescents has become a significant global public health challenge (1). The high incidence of type 2 diabetes mellitus among pediatric offspring of affected parents is attributable not only to genetic predisposition but also to adverse familial lifestyle factors (2, 3). Evidence suggests that children with one parent diagnosed with diabetes have a 15% risk of developing type 2 diabetes mellitus, whereas this risk increases to 75% among those with both parents affected (4). This susceptibility represents a two to fourfold higher risk of the disease compared to offspring of parents without diabetes (5, 6). Persistent hyperglycemia and metabolic dysregulation can trigger a spectrum of diabetic complications, including diabetic cardiomyopathy, nephropathy, and neuropathy, which adversely affect multiple vital tissues and organs (7). Diabetes is not only a personal health crisis but also a significant global socioeconomic challenge. The extensive scope and profound impact of this disease make it an urgent public health priority. The International Diabetes Federation (IDF) advocates for increased investment in targeted preventive healthcare initiatives to enable the early identification of high-risk populations, thereby delaying or preventing the onset of type 2 diabetes mellitus and its associated complications (8). A healthy lifestyle is the most effective strategy for preventing or delaying pediatric type 2 diabetes. In this context, an accurate parental perception of their children's risk of diabetes is a fundamental prerequisite for creating a health-promoting family environment (9–11). In the field of health and disease research, Blalock et al. (12) first introduced the concept of perceived disease risk, which refers to individuals' subjective assessment of their own susceptibility to a specific disease. This perception serves as a key predictor influencing health behaviors and self-management abilities. Children and adolescents have limited self-management abilities due to their immature neurocognitive development and underdeveloped social functioning. They primarily rely on parents and caregivers to maintain healthy behaviors, and the caregivers' risk perception plays a crucial role in influencing health behavior changes in children and adolescents (13). Therefore, understanding the current perceptions of parents with diabetes about their minor children's risk of developing the disease is crucial for promoting healthy lifestyles in the next generation. Existing research has primarily focused on quantitative studies examining risk perception among individuals with diabetes, those at high risk for the disease, and adult offspring of diabetic parents (14–17). However, qualitative studies examining the subjective risk perception of parents with type 2 diabetes mellitus concerning their minor children remain scarce. In this study, parental perception of diabetes risk is conceptualized using the Health Belief Model (HBM) (18). It refers to type 2 diabetic parents' subjective cognitive evaluation of their minor children's genetic susceptibility, disease severity, potential psychological concerns, and willingness to engage in preventive health behaviors. Addressing this research gap, the present study employed a phenomenological approach to explore the current state of diabetes risk perception among affected parents of minor children. The findings aim to provide theoretical foundations and practical implications for developing targeted early intervention strategies for pediatric diabetes prevention.

## 2 Methods

### 2.1 Study design

This study employed phenomenological qualitative design.

### 2.2 Participants and sampling

This study employed purposive sampling using a maximum variation sampling strategy (19). Participants were parents diagnosed with type 2 diabetes mellitus who had minor children. They were recruited from both outpatient and inpatient settings in the Department of Endocrinology and Metabolism at a tertiary hospital in Zhenjiang. The recruitment period spanned from June to August 2025. We examined how differences among various populations might influence research outcomes. To gain deeper insights into the subject, we focused on factors such as role identity, children's age groups, educational attainment, disease duration, family history, and the presence of diabetes-related complications. The inclusion criteria were as follows: Participants met the World Health Organization (WHO) diagnostic criteria for type 2 diabetes mellitus and had a disease duration of at least 12 months; Participants had one or more children, with at least one child under 18 years of age; Participants possessed fluent verbal expression and adequate communication skills to clearly articulate their personal thoughts and feelings; Participants voluntarily agreed to participate in this study and provided written informed consent. The exclusion criteria were as follows: Participants whose minor children had been diagnosed with diabetes or prediabetes; Participants who did not live with their minor children. The sample size was determined by continuing data collection until no new themes emerged (19). Data saturation was assessed continuously throughout the interview process. After interviewing 14 eligible participants, no new themes emerged. To rigorously confirm saturation, two additional qualified participants were subsequently recruited and interviewed. These supplementary interviews yielded no new codes or themes, confirming that adequate data saturation was achieved in this study.

### 2.3 Survey contents

The semi-structured interview protocol was developed based on the research objectives, Health Belief Model and relevant existing literature (18, 20, 21). Its content validity was evaluated by a panel of experts specializing in chronic disease management and qualitative research methodology. Minor modifications were made to ambiguous and repetitive items in accordance with the experts' feedback. Additionally, pre-interviews and group discussions were conducted to revise and optimize the initial interview outline. All ambiguous, repetitive, and inappropriate items were further adjusted and refined, resulting in the finalized formal interview protocol.

### 2.4 Data collection

Prior to each formal interview, the researcher explained the research purpose, significance, and confidentiality principles to all participants. Formal interviews commenced only after participants fully understood this information, provided verbal consent, and established effective rapport with the researcher. All interviews were conducted privately, either in the diabetes health education outpatient clinic or the ward conference room, with no additional personnel present to ensure the authenticity and confidentiality of the interview data. The researcher was a female nurse from the Endocrinology and Metabolism Department, holding a master's degree and having undergone systematic training in qualitative research methods. She conducted all the interviews. After obtaining written informed consent from the research participants, the interviews were recorded. Participants were informed that the recordings would be used exclusively for this study to protect their privacy. Throughout the interview process, the researcher maintains a neutral and objective stance, refraining from guiding or prompting the patient's responses. The patient is encouraged to openly and thoroughly express their perspectives and emotions. Techniques such as probing and clarification are employed to ensure an accurate and comprehensive understanding of the patient's responses. To ensure the completeness and accuracy of the interview data, the entire interview process was audio-recorded, and the recordings were promptly transcribed after each session. Each interview lasted 30 to 40 min, and no participants withdrew from the study.

### 2.5 Data analysis

Data analysis employed Colaizzi's seven-step method (19), with the key steps as follows: familiarize with the raw data: the researchers repeatedly and carefully read all participants' interview transcripts, taking notes to develop an overall perception and a preliminary understanding of the content. Identification of meaningful statements: Meaningful statements related to children's disease risk were extracted, and their corresponding research phases were annotated. N13: “I got diabetes mainly because of my poor diet, but my daughter has healthy eating habits and is not overweight at all.” (Cognitive bias of disease risk) Formulation of meanings: All extracted meaningful statements were coded, and their core implications were summarized and refined. N2: “I have this disease, and my son will definitely get it sooner or later; I am just worried about when he will develop diabetes.” (genetic inheritance-genetic determinism- cognitive biases in the perception of genetic risk). Categorizing codes into theme: Two researchers independently identified shared concepts and attributes to develop themes and thematic clusters. NVivo 12.0 (QSR International Pty Ltd, Burlington, MA, USA) software was used to perform clustering analysis on the different themes. Development of comprehensive theme descriptions: Each extracted theme was elaborated in detail to interpret the underlying structure and characteristics of the phenomenon. Distinction and integration of similar perspectives: Similar viewpoints were summarized and further abstracted into higher-level thematic concepts. For example, genetic fatalism and gender bias in risk cognition were integrated into the overarching theme of cognitive biases related to genetic risk. Likewise, genetic determinism and gender bias in risk perception were combined under the broader theme of cognitive biases in the perception of genetic risk. Validation of results: The preliminary analytical results were returned to the original participants for member checking and confirmation. Throughout the data analysis process, two researchers worked independently. When disagreements arose, they were resolved through team discussions.

### 2.6 Rigor

The rigor of the research process was enhanced by applying the evaluation criteria proposed by Lincoln and Guba in 1985 (22), as follows: (1) Enhancing credibility: the researcher personally worked in the inpatient ward and outpatient clinic of the Department of Endocrinology and Metabolism, thereby gaining in-depth access to the research setting. A strong and trusting relationship was established with some participants through face-to-face communication and by providing assistance during their clinical visits. Regular discussions were held with research team members—including one postgraduate supervisor, three diabetes specialist nurses, and two nursing postgraduates—throughout data collection and analysis to minimize the researcher's subjective bias. (2) Enhancing dependability and confirmability: during the interviews, the researcher listened attentively to the participants' responses and provided appropriate feedback, maintaining neutrality without affirming or criticizing their answers. The coding of the transcribed texts was conducted independently by the researcher and an experienced qualitative researcher to ensure consistency and reduce bias. (3) Ensuring transferability: a maximum variation sampling strategy was employed to enhance the richness and diversity of the results. This study adhered to the Consolidated Criteria for Reporting Qualitative Research (COREQ) guidelines for qualitative research reporting (23).

### 2.7 Ethical consideration

Ethical approval for this study was obtained from the Ethics Committee of the Affiliated Hospital of Jiangsu University (Approval No.:KY2025H0717-02). Throughout the research process, the research team adhered to the Declaration of Helsinki to safeguard the rights and interests of all study participants.

## 3 Results

### 3.1 Participants' demographics

After interviewing 14 research subjects without identifying any new themes, two additional subjects meeting the criteria were included to confirm whether data saturation had been achieved. Ultimately, this study incorporated 16 research subjects, whose basic characteristics are presented in Table 1.

**Table 1:** Description of study participants (*n* = 16).

Number	Role	Age (years)	Disease duration (years)	Educational level	Family history	Complications	Children gender	Age of children
N1	Father	43	6	High school degree	No	No	Girl	15
N2	Mother	37	3	High school	Yes	No	Boy	8
N3	Father	36	2	College degree	No	No	Boy	1
N4	Father	39	1	High school degree	Yes	No	Boy	9
N5	Father	51	6	Master's degree	Yes	No	Boy	11
N6	Father	54	6	College degree	No	No	Boy	17
N7	Mother	55	3	Junior high school degree	Yes	Yes	Boy	16
N8	Mother	46	6	High school degree	No	No	Boy	17
N9	Mother	44	15	Junior high school degree	Yes	Yes	Girl	13
N10	Father	45	2	High school degree	Yes	No	Girl	14
N11	Father	43	1	College degree	No	No	Girl	17
N12	Mother	46	8	Junior high school degree	No	No	Girl	16
N13	Mother	39	9	Primary education	No	No	Girl	17
N14	Father	41	8	College degree	No	No	Boy	15
N15	Mother	38	14	College degree	Yes	No	Boy	14
N16	Father	28	1	College degree	Yes	No	Girl	2

### 3.2 Theme 1: cognitive biases in the perception of genetic risk

#### 3.2.1 Genetic determinism

Some parents with type 2 diabetes hold the belief that parental diabetes inevitably leads to the onset of the disease in their children. This perception is even stronger among families with multiple diabetic relatives, leaving them with an inescapable sense of fatalism.

I have this disease, and my son will almost certainly inherit it. I am now worried about when he will begin to show symptoms. (N2)

My parents and sister all have diabetes. My child will likely develop it in the future—it's probably genetic. Sigh, I feel like I've done my child a disservice. (N7)

#### 3.2.2 The concept of acquired illness leads to cognitive impairment

While some parents with diabetes have a relatively clear understanding of the genetic risk and recognize that their children have a higher likelihood of developing the disease compared to the general population, those who are the first generation in their family to have diabetes tend to underestimate this genetic risk.

I understand that diabetes can be hereditary, but I developed it later in life. My blood sugar spiked because I frequently stayed up late, consumed sugary drinks, and led an irregular lifestyle—neither of my parents has this disease. Therefore, the likelihood of my child developing it should be quite low. (N6)

I am the only person in my family with diabetes, so I believe my child will never develop it. (N5, N14, and N16)

#### 3.2.3 Gender bias in risk perception

Some parents with diabetes hold gender-biased perceptions of genetic risk, believing that female offspring have significantly lower genetic susceptibility to diabetes due to their lower caloric intake and greater awareness of body weight management.

I have a daughter, so I'm lucky—her chance of getting it is slim. Girls are obedient, and she's not like me at all; she doesn't eat much either. (N10)

I don't worry much about my daughter. She cares a lot about her appearance, so she usually takes good care of herself and maintains a healthy weight. (N12)

I developed this disease by eating recklessly, consuming whatever I wanted. My daughter doesn't eat that way, and she isn't overweight either. (N13)

### 3.3 Theme 2: the double-edged sword of behavioral changes following risk perception

#### 3.3.1 Internalization of rational health behaviors

When parents recognize their genetic susceptibility to diabetes, they often take the initiative to adopt lifestyle interventions for themselves, creating an intergenerational demonstration effect of healthy behaviors. This, in turn, promotes the internalization of healthy behavioral patterns in their offspring, ultimately achieving the dual goals of reducing the offspring's risk of developing the disease and optimizing their long-term health outcomes.

I likely developed high blood sugar because I was overweight, and my child is also somewhat heavy. Therefore, I started jogging with her, and we have both gradually lost weight. (N11)

I've cut out sugary drinks now, and my child doesn't drink them much anymore either. (N4)

My son was a bit overweight, so I started working out with him. Now, he can do nearly twenty pull-ups in a row. (N15)

#### 3.3.2 Governance of excessive health behaviors

To reduce their children's risk of developing diabetes, parents with diabetes closely monitor their children's lifestyles and implement various intervention measures. They strictly control their children's diets by limiting the intake of sugary and high-calorie foods and regularly monitor their children's blood glucose levels. However, this increased attention and intervention can sometimes strain family relationships.

The last time I took my child to the hospital, the doctor said they were suffering from malnutrition. Everyone in the family blamed me for being too strict and not allowing the child to eat enough. I was afraid to give them food because I was scared. Now, I truly don't know how to manage my child's diet anymore. (N2)

I'm just worried that her blood glucose levels will be high, so I check it for her every day. Now, she absolutely refuses to let me do it, won't listen to anything I say, and even has major arguments with me. She's deliberately acting against me now, but I'm only doing this for her own good. (N9)

### 3.4 Theme 3: dilemmas in health behavior intervention following risk perception

#### 3.4.1 Intergenerational conflict

In terms of the health management of their children, there are significant intergenerational conflicts in health perceptions among family members across generations. Parents with diabetes advocate the implementation of structured health supervision, while grandparents cling to traditional parenting concepts. This division of intergenerational health authority makes it difficult for children to establish healthy lifestyles.

We pay close attention to our child's diet and ask her to reduce her intake of sugary drinks, milk tea, and sweets. We rarely buy these items for her now. However, the older adults family members cannot bear to see the child unhappy and secretly purchase these treats behind our backs. Despite our repeated discussions, they refuse to listen to us. We truly have no idea what to do. Sometimes, this leads to disagreements and quarrels. (N1)

The older adults family members at home also argue with me about this. They say the child is in a critical period of growth and development and accuse me of not allowing him to eat or drink anything, as if I am harming him. I am his biological mother—how could I not want the best for him? (N2)

When we take care of the child ourselves, we usually cook with cooking oil specially formulated for children. However, when the older adults take care of him, we have to follow their cooking methods. They don't listen to us, and we feel helpless! (N3)

The older adults at home are set in their ways and dote on their grandson very much. They let him eat whatever he wants and never intervene. Sometimes, they even say we are overreacting. (N7)

His grandparents can't bear to refuse him anything. They buy him whatever he wants to eat. It's impossible to reason with them, and we have no control over the situation. (N8)

#### 3.4.2 Conflict between academic load and health maintenance

Some parents with diabetes have adolescent children who are in a critical developmental stage marked by mounting academic pressure and the formation of healthy behavioral patterns. When academic demands conflict with healthy behaviors, most parents, prioritizing their children's studies, tend to adopt a temporary compromise strategy toward their children's health-risk behaviors such as poor dietary habits and a sedentary lifestyle.

My child is currently under a lot of pressure from school right now. I want her to go out and exercise, but she stays up late every night doing homework, so we have never been able to stick with it. (N1)

He's still overweight, but you can't push him too hard given the immense pressure of high school. Academics are the top priority at this stage, which is why we have never fully committed to making him lose weight. (N7)

#### 3.4.3 Scarcity of health management information

After recognizing the genetic risk of diabetes in their children, parents with the disease intend to reduce their kids' risk of developing it through lifestyle interventions. However, they struggle to take effective action due to insufficient preventive health knowledge.

To be honest, we don't fully understand this area. We hope to acquire relevant knowledge to guide us in managing our child's health. (N1)

I hope someone can advise us on how to prevent our child from developing diabetes. (N7)

#### 2.4.4 Game of health sovereignty

Faced with the issue of genetic diabetes, some parents have begun to manage their children's physical health to prevent them from developing the disease. However, the parents' monitoring behaviors driven by a protective motive are often perceived by their children as an infringement of their autonomy.

I tell him to go outside and exercise, but he just won't listen. Kids are so hard to manage these days—you can't communicate with them properly, and they just don't understand. (N8)

Sigh, I've talked to him about it, but it's useless. He's a bit overweight to begin with, has a very irregular diet, and just loves fried food and sugary drinks. Talking to him about it does no good. As soon as you bring this up with him, he starts arguing with you, thinks we're nagging, and says we're too overbearing. (N7)

## 4 Discussion

This study systematically explored type 2 diabetic parents' risk perceptions and corresponding behavioral responses regarding their children's susceptibility to type 2 diabetes. This study revealed that most parents with type 2 diabetes mellitus are aware of the hereditary nature of diabetes; however, their perceptions of genetic disease risk remain inconsistent and varied. Some parents exhibit a tendency toward genetic determinism, believing that their children are inevitably predisposed to diabetes while overlooking the crucial regulatory role of environmental factors in disease development. This result is consistent with the findings of Pih1 et al. (24). Healthcare professionals are advised to deliver targeted health education to parents with type 2 diabetes about the genetic risks of diabetes and the importance of prevention. They should emphasize that maintaining a healthy lifestyle can effectively prevent or delay the onset of the disease in their children. In contrast, some parents in this study exhibited selective risk avoidance, holding biased views such as “diabetes is acquired and non-heritable” and “female offspring with healthy body shape can avoid diabetes.” From the perspectives of cognitive psychology and the Health Belief Model (18), this subjective underestimation of disease risk arises not only from insufficient health literacy but also serves as a psychological defense mechanism to alleviate intergenerational guilt associated with the hereditary transmission of parental type 2 diabetes (25). The common belief that females who maintain an optimal body weight are immune to diabetes warrants careful scrutiny. Although epidemiological studies indicates a generally lower incidence of diabetes among females compared to males (26), no credible evidence supports the notion that females with a family history of diabetes can entirely avoid the risk of the disease solely by maintaining an ideal body weight. This study further enriches the theoretical understanding of family-based genetic risk perception. Our study demonstrates that threat appraisal in the context of familial chronic diseases is influenced not only by objective disease knowledge but also significantly modulated by negative emotional factors, including guilt and anxiety. Among diseases with known genetic susceptibility, affected relatives can play a crucial role in encouraging unaffected family members to adopt preventive measures (27, 28). Relevant studies have indicated that the offspring of patients with diabetes generally acquire knowledge about how to reduce diabetes risk from family members (29). However, more than 55% of patients with diabetes fail to recognize the importance of communicating information related to genetic risks to their family members (30, 31). Therefore, it is crucial to guide parents with diabetes in developing a scientific understanding of genetic risk, enabling them to fully comprehend their children's likelihood of developing the disease and to implement timely and effective preventive measures. Additionally, it is important to address the underlying guilt that these parents may experience and to emphasize the importance of proactively communicating genetic risk information to safeguard their children's health.

As is well known, even if an individual has a genetic predisposition (32), diabetes is largely preventable. The key mechanism involves modifying risky behaviors, such as physical inactivity and unhealthy diet; regulating these behaviors helps reduce the risk of diabetes (33). Protection Motivation Theory (PMT) posits that individuals with higher risk perception are more likely to adopt protective health behaviors (34). Nevertheless, the present study further identifies a potential adverse consequence of excessively heightened risk perception. Rather than continuously promoting adaptive health practices, extreme risk appraisal may trigger maladaptive psychological and behavioral responses, thereby yielding counterproductive outcomes for family-based disease prevention. This finding is consistent with the results reported by Park et al. (35). This study found that although some parents with diabetes recognize their children's risk of developing the disease and have implemented healthy behavioral interventions to prevent it, the effects of these interventions vary significantly. Teh et al. (36) found that risk perception may be influenced by various factors, such as personal experience, family medical history, and the amount of health information obtained, which leads to differences in the adoption of healthy behavioral changes. Patients with a family history of diabetes exhibit greater awareness of disease risk and engage in more preventive behaviors than those without such a history (37, 38), consistent with the findings of the present study. This study found that parents with diabetes who have a family history of the disease or its complications exhibit a heightened perception of risk. However, their excessive vigilance regarding their children's health can lead to adverse consequences. This phenomenon may be attributed to insufficient health literacy regarding diabetes prevention, which leads parents to implement excessive interventions in their children's daily diet and physical activity monitoring in an attempt to reduce their offspring's risk of developing the disease. However, excessive parental attention and intervention can impose significant psychological pressure on children, negatively impacting their physical and mental wellbeing and hindering the development of independence. Therefore, healthcare professionals should support parents with type 2 diabetes in balancing their children's health management with the promotion of independent development. Targeted assessments of children's diabetes risk and personalized preventive guidance are essential to enable rational and evidence-based family-centered diabetes prevention.

Type 2 diabetes has a clear genetic predisposition and often clusters within families. The emerging strategy for diabetes prevention focuses on empowering individuals with diabetes to serve as family health educators. This model facilitates the transmission of health knowledge across generations and ultimately enhances the effectiveness of early preventive initiatives (30). Studies have shown (39) that the intimacy between family members and patients with diabetes influences risk perception. When individuals with diabetes directly influence the health behaviors of their family members, those relatives tend to exhibit stronger motivation for self-management. This finding, however, contradicts the results of the present study. This study found that parents with diabetes face multiple barriers when implementing health behavior interventions for their children, with family-related resistance being particularly prominent. In terms of family relationships, parental interventions regarding the dietary and lifestyle habits of children with diabetes can trigger conflicts among three generations of family members. These conflicts not only hinder effective health management of children by parents with diabetes but also negatively affect the children's development and family harmony. This may be linked to a lack of disease-related knowledge among family members (21). It is imperative to implement structured health education for families affected by diabetes, empower them to develop independent capabilities for prevention and control, and establish a sustainable support system for effective disease management. In addition, the conflict between children's academic performance and health management arises from parents with diabetes rigidly adhering to the perception of a binary opposition between academics and health. Healthcare professionals must reconstruct this cognitive framework and incorporate health management practices into the learning process, thereby transforming health management into a catalyst for academic success.

### 4.1 Limitations

This study has several limitations that should be acknowledged. First, all participants were recruited from the outpatient and inpatient departments of the Endocrinology and Metabolism Division at a single tertiary-level hospital in Jiangsu Province, which leads to certain sampling constraints. The single-center sampling setting may restrict the diversity of participants' socioeconomic backgrounds, medical access experiences, and health cognition levels. Second, the study findings are influenced by the local cultural context and regional healthcare service characteristics. The health beliefs, family health concepts, and medical service utilization patterns of diabetic parents in Jiangsu may differ from those in other regions of China, due to variations in regional living habits, health education popularization, and family parenting concepts. Additionally, the regional healthcare system arrangement, including hierarchical diagnosis and treatment, chronic disease management policies, and community health service coverage, may further influence parents' risk perception and health decision-making, which limits the cross-regional applicability of the results. Third, consistent with the characteristics of qualitative research, this study focuses on in-depth exploration of individual experiences rather than universal generalization. Therefore, the transferability of the findings is restricted, and the research conclusions cannot be directly extrapolated to diabetic parents in other economic, cultural, and medical environments. Future studies could broaden the sampling scope by enrolling diabetic parents from diverse geographical regions and multiple levels of medical institutions, with an increased sample size. Additionally, further investigation into the effects of cultural characteristics and disparities in healthcare systems on parental disease cognition and health-related behaviors is warranted to enhance the reliability and generalizability of the research findings.

## 5 Conclusions

This phenomenological qualitative study explored parents' perceptions of type 2 diabetes risk among their minor children. Three core themes were identified: cognitive biases in the perception of genetic risk, the double-edged sword of behavioral changes following risk perception, and dilemmas in health behavior intervention following risk perception. The present findings can be generalized to broader social and public health contexts, offering valuable insights for family-centered diabetes prevention and population-based health promotion strategies. Healthcare professionals can develop targeted intervention programs tailored to parental risk perception characteristics to enhance the prevention of pediatric type 2 diabetes, thereby delaying or preventing disease onset in this high-risk offspring population.

## Data Availability

The raw data supporting the conclusions of this article will be made available by the authors, without undue reservation.
