# Supplementary material for: The perception of diabetes risk in minor children among parents with type 2 diabetes mellitus: a qualitative study
Source: Front Public Health. 2026 Jun 4;14:1846450. doi: 10.3389/fpubh.2026.1846450 (PMC13275426; doi:10.3389/fpubh.2026.1846450)
Supplement: Supplementary file 1 [file Table_1.docx]

**Appendix 1**

**Interview outline**

| **Q1** | How long have you been suffering from diabetes? |
| --- | --- |
| **Q2** | Are you aware of the genetic risk of diabetes? What do you think about the heredity of diabetes? |
| **Q3** | Are you worried that your diabetes will be inherited by your children? Why or why not? |
| **Q4** | What measures have you taken to address this concern? |
| **Q5** | What obstacles have you encountered in the process of taking these measures? |
